# Rapid Monitoring of Storage Deterioration in Processed Coix Seeds Using Near-Infrared Spectroscopy Guided by GC–IMS

**DOI:** 10.3390/foods15091542

**Published:** 2026-04-29

**Authors:** Jiangshan Zhang, Tongtong Wu, Xiangyang Yu, Ming Yang, Penghui Zeng, Xiaolin Xiao, Yushan Li

**Affiliations:** 1 Key Laboratory of Modern Preparation of Chinese Medicine, Ministry of Education, National Key Laboratory of Classic Formula and Modern Traditional Chinese Medicine Creation, Jiangxi University of Chinese Medicine, Nanchang 330004, China; zjs555126@163.com; 2 State Key Laboratory of Optoelectronic Materials and Technologies, School of Physics, Nanchang Research Institute, Sun Yat-sen University, Guangzhou 510275, China; cesyxy@mail.sysu.edu.cn; 3 Jiangxi Jingde Traditional Chinese Medicine Co., Ltd., Jingdezhen 333000, China; 18288200504@163.com (P.Z.);

**Keywords:** coix seed, near-infrared spectroscopy, gas chromatography–ion mobility spectrometry, lipid oxidation, storage quality, rapid non-destructive monitoring

## Abstract

Processed coix seeds are widely consumed as both food and traditional medicinal materials, but their quality gradually deteriorates during storage due to lipid oxidation and rancid odor formation. In this study, volatile changes during storage were characterized using gas chromatography–ion mobility spectrometry (GC–IMS), and a rapid monitoring method based on near-infrared spectroscopy (NIRS) was developed. GC–IMS identified 74 volatile compounds, with aldehydes and ketones increasing significantly during storage, indicating progressive lipid oxidation. Key markers, including 2-furaldehyde, 1-pentanoic acid, and γ-caprolactone, were identified as indicators of quality deterioration. Based on these markers, composite flavor and storage deterioration indices were constructed and used as reference parameters for NIRS calibration. Partial least squares regression models developed in the 1300–2500 nm region showed strong predictive performance for these composite indices, with $R^{2}$p > 0.93 and RPD > 4.0. The long-wave NIR region exhibited superior sensitivity to oxidation-related spectral changes. These results demonstrate that NIRS combined with GC–IMS analysis provides an effective, chemically interpretable approach for rapid, non-destructive monitoring of storage quality in processed coix seeds.

## 1. Introduction

Coix seed is the dried mature seed kernel of *Coix lacryma-jobi* L. var. *ma-yuen* (Roman.) Stapf, a plant belonging to the Poaceae family [1,2]. Modern research indicates that coix seed possesses various pharmacological activities, including antitumor, antiallergic, immunomodulatory, and gut microbiota regulatory effects [3]. It is commonly used to treat conditions such as edema, beriberi, dysuria, and diarrhea [4,5]. In traditional medicine, coix seeds are often processed through methods such as steaming, sun-drying, or roasting to produce processed coix seeds. These processed seeds exhibit a popcorn-like appearance with a roasted aroma, which enhances their efficacy in strengthening the spleen and stopping diarrhea. Moreover, they can be consumed directly without prolonged boiling, highlighting their considerable potential for both medicinal and dietary applications.

After processing, coix seeds exhibit enhanced pharmacological efficacy and improved palatability. However, they are highly susceptible to developing rancid off-odors during prolonged storage. Studies have shown that low-molecular-weight aldehydes and ketones generated via lipid oxidation are major contributors to rancid odor formation, reflecting internal chemical deterioration processes [6]. This process not only reduces consumer acceptability and patient compliance but may also compromise medicinal efficacy and edible quality. Currently, the evaluation of rancid odor in processed coix seeds primarily relies on human sensory assessment, which is subjective and poorly reproducible [7]. Previous studies have mainly focused on lipid oxidation mechanisms in coix seeds [8] and changes in volatile compound profiles before and after processing [9]. However, systematic investigations into the dynamic variation of flavor compounds in processed coix seeds during storage, as well as the development of rapid and objective evaluation techniques, remain limited.

In recent years, NIRS technology has been extensively applied in studies examining the storage duration and quality assessment of various Chinese herbal medicines, including identifying the storage duration of nuts [10], authenticating the origin of Chinese herbal medicines [11], verifying the authenticity of Cuscutae Semen [12] and detecting adulteration in Lonicerae Japonicae Flos [13]. The vibrational absorption characteristics of NIRS associated with C-H, O-H, and N-H functional groups enable indirect monitoring of dynamic changes in chemical constituents potentially related to rancid odor formation [14]. However, NIRS has limited capability for direct identification of specific chemical compounds. Its main advantages include rapid, non-destructive, on-site detection and relatively low analytical cost. Meanwhile, GC–IMS, as a highly sensitive technique for visualization and separation of volatile compounds, has been widely applied to investigate processing- and storage-induced changes in volatile flavor profiles of Chinese herbal materials and grains [15]. GC–IMS provides compound-level chemical information that can enhance the interpretability of NIRS models, though it typically requires longer analysis time and higher operational cost. Therefore, integrating the complementary strengths of both techniques offers a promising approach to construct a quantitative spectrum–flavor correlation model, enabling rapid on-site detection while establishing objective chemical evaluation metrics.

The objective of this study was to develop a chemically interpretable and rapid monitoring strategy for storage deterioration in processed coix seeds. Unlike conventional NIRS modeling approaches that rely solely on spectral–property correlations, this study introduces a chemically guided calibration framework by incorporating GC–IMS-derived volatile markers into model construction. By establishing a composite deterioration index reflecting key chemical changes, the proposed approach aims to improve both predictive performance and model interpretability. This strategy provides a more mechanistically meaningful link between spectral features and underlying quality changes during storage.

## 2. Materials and Methods

### 2.1. Samples Preparation

**Figure 1:**
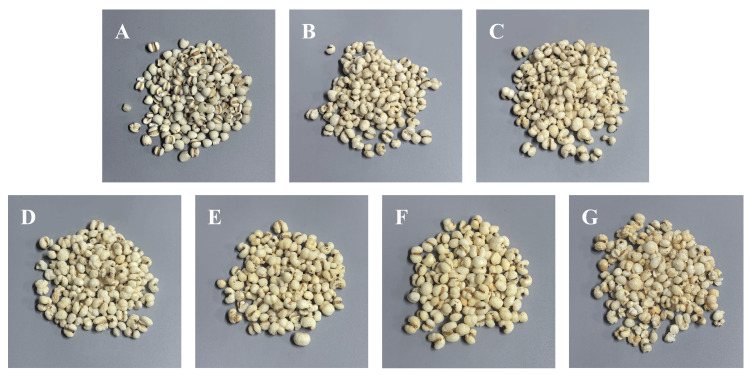
Images of coix seed samples: (**A**) S-Raw sample; (**B**) P2-Processed sample stored for 2 months; (**C**) P4-Processed sample stored for 4 months; (**D**) P12-processed sample stored for 12 months; (**E**) P29-processed sample stored for 29 months; (**F**) P46-processed sample stored for 46 months; (**G**) P56-processed sample stored for 56 months.

The coix seed samples used in this study were supplied by Jiangxi Jingde Chinese Medicine Co., Ltd. (Jingdezhen, Jiangxi Province, China). All samples were derived from the same batch of raw material to minimize variability unrelated to storage conditions and included raw seeds and six groups of processed seeds stored for different durations. Processed coix seeds were prepared from raw seeds following the processing procedure specified under the “Coix Seed” entry in the Jiangxi Provincial Standard for Processing Chinese Herbal Medicinal Materials [16]. This procedure, which involves sequential steps of soaking or boiling, steaming, drying, and stir-frying with hot sand, effectively removes excess free water and yields seeds with a uniformly low and stable moisture content prior to storage. The processed coix seeds exhibited characteristic features, including a light-yellow surface, numerous puffed or cracked grains, and a typical roasted aroma characteristic of processed coix seeds, as shown in Figure 1.

The raw coix seed sample was designated as S, whereas processed samples were labeled according to storage duration in months as P2, P4, P12, P29, P46, and P56, where the numbers indicate the number of months the samples had been stored. All coix seed samples were stored in a moisture-proof cabinet under controlled laboratory conditions at approximately 20 °C. Samples were sealed with desiccants to maintain a stable low-moisture environment and minimize interference from external moisture and ambient odors. This controlled storage protocol was specifically implemented to suppress moisture variability as a confounding factor in subsequent NIRS measurements, ensuring that spectral differences among samples with varying storage durations primarily reflect chemical transformations such as lipid oxidation rather than fluctuations in water content.

### 2.2. NIRS Analysis

#### 2.2.1. Instrumentation and Spectral Data Acquisition

Two portable handheld near-infrared spectrometers manufactured by Guangzhou Opt-Info Technology Co., Ltd. (Guangzhou, Guangdong, China) were used in this study: GX-IRSV-TEC10 and GX-I-S1325. The GX-IRSV-TEC10 spectrometer operates in the spectral range of 900–1700 nm, providing 228 wavelength points, and was equipped with two 0.7 W tungsten-halogen lamps. The GX-I-S1325 covers a spectral range of 1300–2500 nm with 257 wavelength points and was also equipped with two tungsten-halogen lamps as light sources. Together, these two spectrometers enable coverage of most of the NIR spectral region. Both spectrometers acquired spectral data in diffuse reflectance mode. Reflectance was calculated using a standard white reference plate, and spectra were converted to absorbance using $\log\left.(\frac{1}{R}\right.)$ transformation.

The spectral data acquisition procedure was performed as follows. The instrument was preheated for 15 min to stabilize the light sources, followed by standard white reference calibration. Each sample was divided into five subsamples and placed sequentially into a sample cup. The sample cup was positioned at the light exit port of the spectrometer. During spectral acquisition, the sample cup was slightly shifted between successive scans to ensure that each measurement captured a different portion of the sample surface, thereby improving representativeness and reducing local heterogeneity effects. A total of 10 spectra were collected for each subsample, resulting in 50 NIR spectra per sample. Finally, the spectrometer was connected to a computer via Bluetooth for spectral data acquisition.

#### 2.2.2. Data Processing

NIRS data are often affected by baseline drift, light scattering, and random noise during measurement. In this study, multiple preprocessing methods were employed to mitigate interference factors in the data, ensuring accuracy and reproducibility. The Min-Max normalization technique effectively reduces the impact of sample-to-sample or measurement condition variations on the model by adjusting data to a uniform scale [17]. To enhance the signal-to-noise ratio and correct scattering effects, four preprocessing methods were applied to raw spectra for comparison: raw spectrum (RAW), Savitzky-Golay filtering combined with first-order differentiation (SGFD), multiple scattering correction combined with first-order differentiation (MSCFD), and standard normal variable transformation combined with first-order differentiation (SNVFD). The Savitzky-Golay smoothing (SG) algorithm was employed to enhance the signal-to-noise ratio of spectral signals by reducing high-frequency random noise while preserving absorption band shapes [18]. Multivariate scattering correction (MSC) and standard normal variable (SNV) transformation are commonly used scattering correction techniques to eliminate additive and multiplicative effects in spectra caused by variations in particle size, packing density, and surface morphology of the solid samples [19]. First-order derivatives are applied to remove baseline drift and enhance spectral features by resolving overlapping absorption bands, which is particularly important for distinguishing C–H and O–H overtones from lipids and oxidation products in the 1300–2500 nm region [20]. The preprocessed data are then utilized for subsequent classification modeling, with the optimal preprocessing scheme determined by comparing model performance across different methods.

#### 2.2.3. NIRS-Based Classification Modeling

To provide a preliminary evaluation of spectral discrimination ability, several classification models, including Support Vector Machine (SVM), k-Nearest Neighbors (KNN), and Random Forest (RF), were explored based on preprocessed NIRS data. SVM effectively classifies data in high-dimensional spaces by capturing key samples while eliminating redundant ones, making it widely used in statistical classification and regression analysis [21]. KNN is a structurally simple classification algorithm that classifies samples by finding the k nearest training samples in feature space and performing majority voting based on their categories [22]. RF is an ensemble learning method that enhances model accuracy and robustness by constructing multiple decision trees; however, it may suffer from overfitting when dealing with a large number of features or imbalanced samples [23]. We divided the preprocessed spectral data into training and testing sets at an 8:2 ratio, and evaluated the model’s generalization capability using five-fold cross-validation on the training set. Model performance was assessed using accuracy, weighted F1-score, and weighted recall, while simultaneously visualizing classification results through a confusion matrix. Ultimately, the optimal model configuration was determined by comparing the overall performance of different preprocessing methods and classification algorithms. However, as the primary objective of this study was quantitative evaluation of storage deterioration, subsequent analysis focused mainly on regression modeling.

### 2.3. GC–IMS Data Processing

#### 2.3.1. Instrument Conditions and Data Collection

Volatile flavor component data acquisition was performed using the FlavourSpec® GC–IMS instrument from G.A.S. GmbH (Dortmund, Germany), with an automatic headspace sampler CTC-PAL 3 (CTC Analytics AG, Zwingen, Switzerland). The operational procedure for this section is as follows: Accurately weigh 5.0 g of coix seed sample, place it in a 20 mL headspace vial, and thoroughly chop. Incubate the sample at 60 °C and 500 rpm for 15 min to promote the migration of volatile components into the headspace. Injection was performed in splitless mode with the injection needle temperature set to 85 °C and an injection volume of 500 µL.

Gas chromatography separation was performed using an MXT-5 capillary column (Restek Corporation, Bellefonte, PA, USA) (15 m × 0.53 mm, film thickness 4 μm) maintained at a constant column temperature of 60 °C. Both carrier gas and drift gas were high-purity nitrogen (purity ≥ 99.999%). The carrier gas flow rate was programmed as follows: an initial flow rate of 2.0 mL/min maintained for 2 min; followed by a linear increase to 10.0 mL/min over 8 min; then a further linear increase to 100.0 mL/min over 10 min, with the flow rate held constant at this value for 10 min. The drift gas flow rate was set at 150 mL/min.

The ion migration spectrometry parameters were set as follows: the ionization source is a tritium source ${(}^{3}H)$, the migration tube length is 98 mm, the electric field strength is set to 500 V/cm, the migration tube temperature is maintained at 45 °C, and detection is performed in positive ion mode. Each sample underwent three parallel measurements to ensure data reproducibility.

#### 2.3.2. Data Processing and Multivariate Analysis

Raw GC–IMS data were processed using VOCal (v0.4.10) software and imported into GC × IMSLibrary Search V2.2.1 for peak identification and qualitative analysis. Prior to multivariate analysis, all peak volumes were normalized to the internal standard 2-octanol-D to correct for injection variability and instrumental drift. 2-Octanol-D was selected due to its stable signal intensity and distinct, interference-free drift time. Single-standard normalization assumes proportional detector responses across volatile compound classes, consistent with established GC–IMS non-targeted flavor profiling practices. Volatility fingerprint profiles were constructed using the GalleryPlot plugin, and differential profiles were employed to compare significant differences among samples stored for varying durations. Processed peak area data were exported and imported into SIMCA-14.0 for multivariate statistical analysis, including Principal Component Analysis (PCA) and Orthogonal Partial Least Squares–Discriminant Analysis (OPLS–DA). Key volatile biomarkers were screened based on VIP values. A composite deterioration index was then derived from the PLSR latent variable scores associated with storage time using the normalized intensities of VIP-selected compounds. Non-retained compounds were either monomer/dimer redundancies or showed no systematic temporal variation, and therefore did not contribute to index construction.

It should be noted that in GC–IMS analysis, certain volatile compounds, particularly carboxylic acids and alcohols, may appear as both monomeric (M) and dimeric (D) ion signals due to ion clustering during the ionization and migration processes [24]. Both signals originate from the same chemical compound but differ in ion aggregation state. In subsequent multivariate analysis, the selection of either the M or D form as a potential marker is influenced by signal intensity, stability, and reproducibility across replicate measurements, rather than indicating chemical differentiation. Throughout this study, when both monomer (M) and dimer (D) ion signals of the same compound were detected, only one form was retained for subsequent multivariate analysis based on signal intensity, stability, and reproducibility across replicates, in order to minimize redundancy and avoid multicollinearity. The consistency of variation trends between M and D forms was systematically evaluated to ensure that the selected signal reliably represented the corresponding compound.

### 2.4. Model Building and Performance Evaluation

Partial least squares regression (PLSR) was employed to construct a chemically guided spectroscopic calibration model. Rather than direct feature-level fusion of GC–IMS and NIRS data, GC–IMS was first used to derive chemically meaningful flavor and deterioration indices, which were subsequently treated as response variables for NIRS calibration. PLSR is a widely used multivariate calibration method that extracts latent variables shared by predictors and responses, thereby maximizing explanatory power while mitigating multicollinearity [25,26].

Initially, a PLSR model was established using GC–IMS peak volume data as predictors and storage time as the response variable. Key compounds were screened based on variable importance in projection (VIP), with VIP >1 as the selection criterion. The composite deterioration index was defined as the latent variable scores derived from a PLSR model constructed with storage time as the response variable (y), integrating the normalized peak volumes of key volatile markers (VIP >1) identified by GC–IMS combined with OPLS–DA into a single continuous, dimensionless metric. These compounds are established products of lipid oxidation and recognized indicators of quality deterioration in cereal grains [27]. The peak volumes of selected compounds were normalized prior to extracting PLSR scores as the composite index. This strategy of constructing composite quality indices from multiple variables and calibrating them against spectral data has been validated in recent food quality studies [28].

In NIRS modeling, the GC–IMS-derived composite index was used as the response variable, with NIRS spectra as predictors. Spectral preprocessing included raw spectra, SG, MSC, SNV, SGFD, MSCFD, and SNVFD, followed by Min–Max scaling. Samples were stratified by storage duration and randomly divided into training (80%) and testing (20%) sets. Five-fold cross-validation within the training set was used to optimize the number of latent variables (LVs, 1–10) by maximizing the cross-validated coefficient of determination ($R_{cv}^{2}$), after which the final model was fitted using all training samples. The held-out testing set, excluded from model development, was used for independent validation within the available cohort. Model performance was evaluated using the coefficient of determination ($R^{2}$), root mean square error (RMSE), and residual prediction deviation (RPD). Generally, RPD >3.0 indicates excellent predictive performance. To further exclude the possibility of overfitting or chance correlation, permutation tests with 200 random iterations were performed for each optimized NIRS–PLSR model. In each iteration, the response variable was randomly shuffled while the spectral matrix remained unchanged, and the resulting $R^{2}$ and $Q^{2}$ values of the permuted models were recorded. A $Q^{2}$ intercept below 0.05 is considered indicative of a statistically robust model free from overfitting. Regression coefficient analysis of the optimal model was performed to identify influential wavelength regions and interpret their chemical relevance.

## 3. Results and Discussion

### 3.1. NIRS Analysis of Coix Seeds

#### 3.1.1. Spectral Feature Analysis

**Figure 2:**
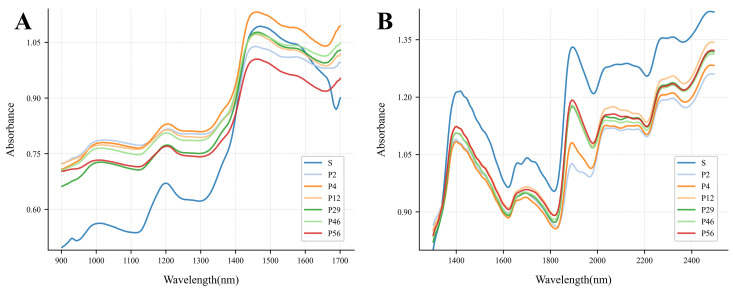
NIR spectra of coix seeds with different storage times: (**A**) 900–1700 nm; (**B**) 1300–2500 nm.

Figure 2 shows the average absorbance spectra of coix seed samples in the short-wave near-infrared (SW-NIR, 900–1700 nm) and long-wave near-infrared (LW-NIR, 1300–2500 nm) regions. Overall, the spectral profiles of different samples are highly consistent, with absorption peaks and troughs occurring in similar wavelength bands, reflecting their chemical similarity. Nevertheless, processing and storage induced noticeable differences in absorbance at specific wavelengths. In the 900–1700 nm range, spectral features mainly arise from second-harmonic and sum-frequency vibrations of O-H, C-H, and N-H functional groups. For instance, the absorption near 1000 nm corresponds to second-harmonic vibrations of hydrogen-containing groups [29]; the trough near 1100 nm and the peak near 1200 nm are attributed to second-harmonic C-H stretching in carbohydrates [30]. In the 1300–2500 nm range, the O–H combination bands near 1420 nm and 1900 nm are extremely strong and could partially mask subtle spectral variations arising from lipid oxidation [31]. However, under the strict moisture control employed in this study, spectral variations in this region are predominantly attributed to oxygen-containing functional groups formed during lipid oxidation. Prior work has linked absorptions near 1697 nm and 1946 nm to C–H and C–O stretching vibrations and ${\mathrm{CH}}_{3}$/${\mathrm{CH}}_{2}$ deformation modes, which effectively track free fatty acid changes in rice [32]. Subtle features near 2300 nm are associated with combination vibrations involving C–H and N–H groups from lipids and proteins [33], whereas weak absorption features near 2100 nm may be related to O–H stretching and combination bands of hydroperoxides, which are primary products of lipid oxidation [34]. The absorption valley near 2200 nm is attributed to combination bands of C–H and O–H groups, reflecting contributions from both lipids and their oxidation products [35]. During prolonged storage of cereals, the progressive accumulation of such oxidation products alters the absorption profiles in these near-infrared regions, providing a reliable spectroscopic basis for non-destructively monitoring the rancidity process of coix seed and discriminating its different storage stages [36]. Consequently, the long-wave near-infrared region generally contains richer chemical information related to storage-induced deterioration and is more suitable for monitoring food quality changes.

Compared with the raw samples, the processed coix seeds exhibited differences in absorbance levels across several characteristic spectral bands. As storage duration increased, the intensity of certain absorption peaks gradually decreased. This phenomenon may be associated with compositional changes such as lipid oxidation, moisture redistribution, and protein modification occurring during storage. These chemical transformations are known to influence both the nutritional stability and flavor quality of cereal products, indicating that NIR spectroscopy can capture spectral signatures related to the deterioration of storage quality in processed coix seeds.

#### 3.1.2. Classification of Coix Seeds by Storage Duration

We conducted a classification study based on the spectral data of seven coix seed samples (S, P2, P4, P12, P29, P46, P56). The data were stratified and randomly split into a training set and a test set at an 8:2 ratio to maintain the class distribution. Modeling and evaluation were performed using combinations of different preprocessing and classification algorithms. The classification results are presented in Table 1. Results indicate that within the two NIRS bands, without preprocessing, the performance of all three classification algorithms (SVM, KNN, RF) was limited, with the highest stratified 10-fold cross-validation accuracy reaching only 0.5710.

Without preprocessing, the performance of the three classification algorithms (SVM, KNN, and RF) was limited in both spectral regions, with the highest stratified 10-fold cross-validation accuracy reaching only 0.571. Classification accuracy improved markedly after preprocessing, indicating that spectral correction enhances the detection of chemical variations associated with storage. Across both spectral ranges, models based on the 1300–2500 nm long-wave NIR region consistently achieved higher accuracies, suggesting that this region is more sensitive to storage-related changes in coix seeds. Among the tested models, the Random Forest (RF) model combined with MSC and derivative preprocessing achieved the best performance, yielding a test accuracy of 0.929 with F1-score and recall values close to 0.93. This strong discrimination ability indicates that NIR spectra capture chemical transformations occurring during storage. In cereal-based foods, prolonged storage is typically accompanied by lipid oxidation, moisture redistribution, and changes in volatile compounds that affect flavor stability and overall product quality. These compositional variations alter vibrational absorption features of molecular bonds such as C–H, O–H, and C=O, which are reflected in the NIR spectra.

Therefore, the successful classification of samples with different storage durations demonstrates that NIRS can effectively detect storage-induced chemical variations in processed coix seeds. To further clarify the chemical basis of these spectral differences, GC–IMS analysis was subsequently performed to characterize the evolution of volatile compounds and identify key markers associated with flavor deterioration during storage.

### 3.2. GC–IMS Analysis

GC–IMS analysis was performed to elucidate the chemical basis underlying the spectral variations observed in NIRS and to identify key volatile compounds associated with storage-induced deterioration. By linking volatile profiles with spectral features, this analysis provides essential guidance for constructing chemically meaningful prediction models.

#### 3.2.1. Volatile Fingerprint Profiles

**Figure 3:**
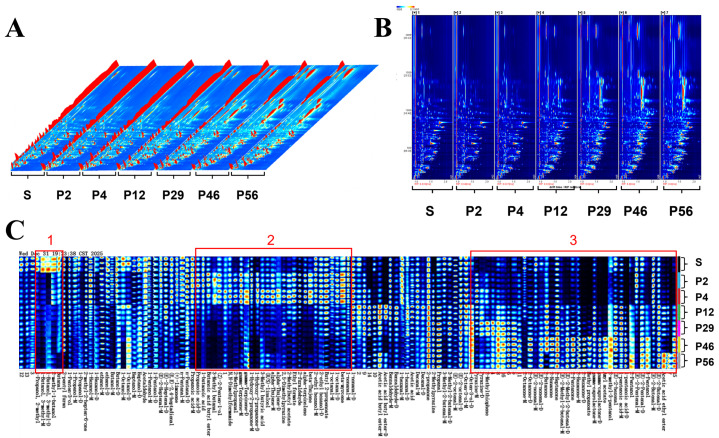
(**A**) Three-dimensional odor spectrum of raw and processed coix seed; (**B**) Two-dimensional odor spectrum of raw and processed coix seed; (**C**) Odor fingerprint spectrum of raw and processed coix seed.

To further explain the storage-related spectral differences observed in the NIR analysis and to identify the chemical components responsible for quality changes, volatile compounds in coix seed samples were analyzed using GC–IMS. The resulting volatile fingerprints revealed clear differences among samples stored for different durations. As shown in Figure 3A,B, GC–IMS effectively separated volatile components across the seven storage stages. Compared with raw coix seeds, processed samples exhibited a noticeable decrease in several original volatile compounds while generating a series of new components. These changes may be associated with thermal reactions occurring during processing, including lipid oxidation and Maillard reactions, which can significantly alter the flavor composition of cereal products.

To further compare volatile profiles, the Gallery Plot was used to generate VOC fingerprints (Figure 3C). Each row represents a sample, and each column corresponds to a volatile compound, with color intensity indicating relative concentration. Clear differences were observed among samples. Compounds in region 1 were abundant in raw coix seeds but markedly reduced after processing, suggesting that these heat-sensitive volatiles represent intrinsic aroma components of the raw material. Compounds in region 2 were mainly generated during processing, showing high concentrations in P2 and P4 but gradually decreasing during storage, indicating their association with processing-induced flavor characteristics. In contrast, most compounds in region 3 increased progressively during prolonged storage and are therefore likely associated with storage-related flavor deterioration, particularly the development of oxidative or rancid odors. These volatile changes provide chemical evidence supporting the storage-dependent spectral variations observed in the NIR analysis.

#### 3.2.2. Identification and Characterization of VOCs

**Figure 4:**
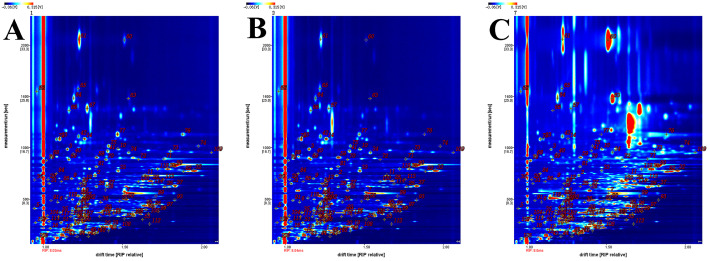
Qualitative analysis of volatile organic compounds in coix seed: (**A**) Sample S; (**B**) Sample P2; (**C**) Sample P56.

We employed GC–IMS to identify and analyze volatile components in different coix seed samples. Figure 4 displays the qualitative spectra for samples S, P2, and P56. Qualitative identification of volatile compounds was based on the NIST 2020 database and IMS database integrated within the GC–IMS Library Search V2.2.1 software. Specific identification results are presented in Table 2. In Figure 4, the horizontal axis represents migration time, while the vertical axis denotes retention time. A total of 74 volatile compounds were identified across the seven coix seed samples, including 19 aldehydes, 18 alcohols, 13 ketones, 9 esters, 6 acids, 3 pyrazines, 2 furans, and several other heterocyclic compounds (Table 2). These compounds mainly originate from lipid oxidation, carbohydrate degradation, and Maillard reactions, which are key processes affecting flavor development during the processing and storage of cereal products. Statistical analysis of peak intensities revealed clear differences in both the distribution and relative abundance of volatile components across storage stages.

Statistical analysis of peak intensities for identified compounds revealed significant differences in the distribution categories and relative abundances of volatile components across storage stages. Overall, aldehyde compounds exhibited the highest total peak intensity, followed by ketones and alcohols, indicating that lipid oxidation is the dominant process forming the rancid odor during coix seed storage. Compared with fresh samples, aldehyde compounds significantly increased in stored samples, exhibiting a gradual upward trend with prolonged storage duration. This pattern aligns with the progressive accumulation of primary and secondary oxidation products during the ongoing oxidation of unsaturated fatty acids. Ketone compounds showed a brief decline during mid-storage before gradually rebounding, indicating the continuous accumulation of secondary lipid oxidation products. Alcohol compounds were abundant in the early storage phase but generally decreased later, possibly due to their further oxidation into aldehydes and ketones or their involvement in esterification reactions. Additionally, acids and esters significantly increased in long-term stored samples, reflecting enhanced fatty acid oxidation and esterification reactions. This caused the sample flavor to gradually shift from fresh grain aroma to oxidative aging characteristics.

GC–IMS analysis revealed that lipid oxidation and non-enzymatic browning reactions jointly drive the evolution of volatile flavors during storage. Different categories of volatile compounds exhibit distinct differences in origin, odor characteristics, and contribution to overall flavor, collectively driving the transition of coix seed flavor from freshness to aging. These findings provide a chemical basis for establishing correlation models based on near-infrared spectral information.

#### 3.2.3. PCA Analysis

**Figure 5:**
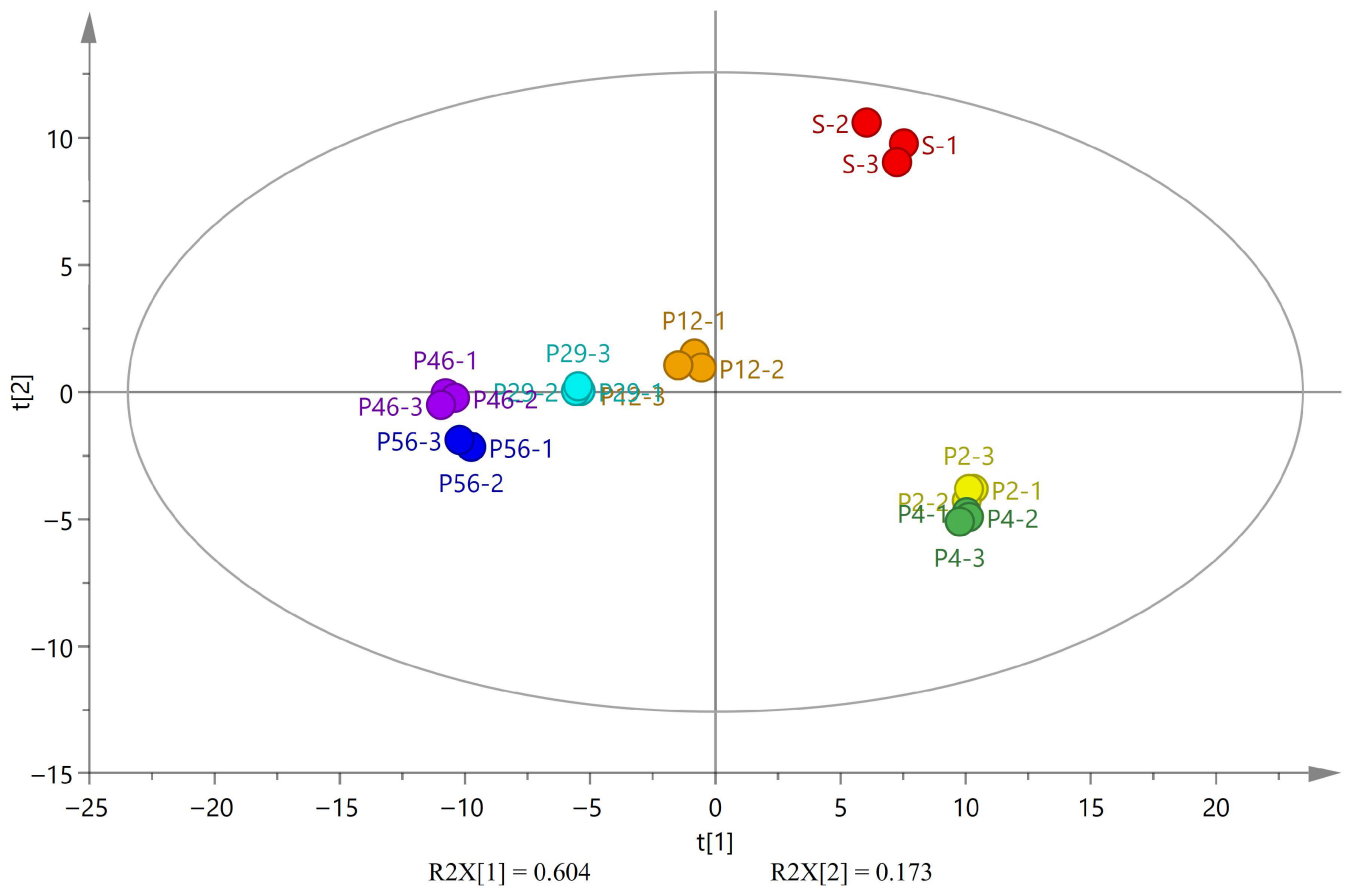
PCA score of volatile components in raw and processed coix seed. PCA: Principal Component Analysis; t1/t2: principal component 1/2; S: raw seed; P2–P56: processed seeds stored for 2–56 months.

Principal Component Analysis (PCA) is a statistical technique used to investigate correlations among multiple variables, effectively identifying relationships between samples while retaining features that contribute most significantly to variance [37]. PCA analysis was performed on the peak volumes of all volatile components measured in this study, with results shown in Figure 5. The contribution rates of t1 and t2 were 60.4% and 17.3%, respectively, with these two principal components accounting for 77.7% of the total variance, which represents the majority of the variance. The seven samples were clearly distinguished and uniformly distributed across four regions, with samples within the same region exhibiting strong correlations. This result indicates significant differences in the overall volatile flavor characteristics of Chinese coix seeds across different storage stages.

The separation between raw and processed samples mainly occurred along the t2 axis, indicating substantial changes in volatile composition after processing. Freshly processed samples (P2 and P4) clustered closely, suggesting relatively stable volatile profiles during the early storage period. In contrast, samples stored for longer periods showed progressive displacement along the t1 axis, reflecting continuous changes in volatile composition during storage. This trend indicates a gradual transition in flavor characteristics from fresh grain notes toward oxidative and aged aromas.

#### 3.2.4. OPLS–DA Analysis

**Figure 6:**
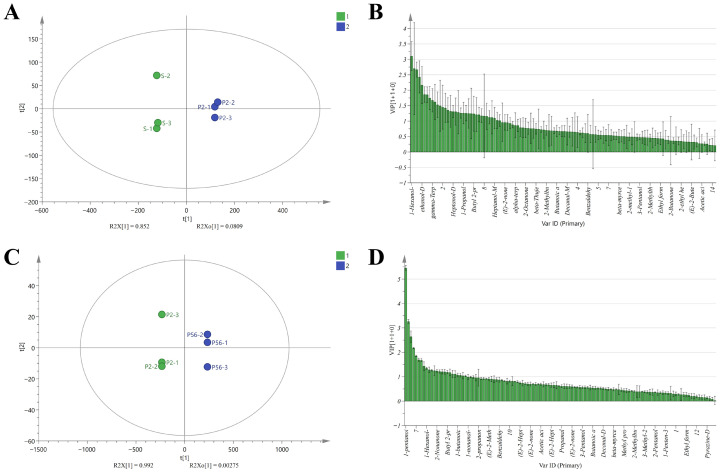
(**A**) OPLS–DA scores for S and P2; (**B**) VIP scores for S and P2; (**C**) OPLS–DA scores for P2 and P56; (**D**) VIP scores for P2 and P56. OPLS–DA: orthogonal partial least squares–discriminant analysis; VIP: variable importance in projection; t1/t2: predictive/orthogonal component scores; S: raw seed; P2/P56: processed seeds stored for 2/56 months.

Based on the results of PCA analysis, we further employed OPLS–DA to perform discriminant analysis on the volatile components of coix seed samples, aiming to identify key differential components during processing and storage. OPLS–DA is a chemometric method based on Principal Component Analysis, designed to eliminate signal interference unrelated to classification and construct a discriminant model between feature variables and sample categories [38]. We employed the peak areas of volatile components from the coix seed S and P2 groups, as well as the P2,56 group, as variables. Using SIMCA-P 14.1 software, OPLS–DA analysis was conducted, with the scatter score plot and VIP score plot shown in Figure 6.

Figure 6A,B present the analysis results for the raw product S and the processed product P2. Figure 6A shows that t1 and t2 contributed 85.2% and 8.1% respectively, totaling 93.3%. The model’s independent variable fit index $R_{X}^{2}$ was 0.933, the dependent variable fit index $R_{Y}^{2}$ was 0.999, the prediction index $Q^{2}$ was 0.991, and the classification prediction accuracy reached 99.1%. Processing significantly influenced the volatile flavor composition of coix seed, and the associated differential signals were effectively captured by the OPLS–DA model. To further identify key biomarkers distinguishing pre- and post-processing samples, 35 volatile components with significant classification contribution (VIP>1) were screened, as detailed in Table 3. Figure 6C,D present the analysis results for processed sample P2 and long-term stored sample P56. As shown in Figure 6C, t1 and t2 contributed 99.2% and 0.28% respectively, totaling 99.4%. The model exhibited an $R_{X}^{2}$ of 0.995, $R_{Y}^{2}$ of 1, $Q^{2}$ of 1, and achieved 100% classification accuracy. While these high metrics reflect pronounced chemical divergence between the compared groups, their interpretation should consider the limited sample size; the identified markers were further supported by consistent temporal trends across all storage stages. The model demonstrates exceptional fitting and predictive capability within this group, indicating that volatile flavor changes induced by long-term storage exhibit strong stage-specificity. To identify key biomarkers of flavor evolution in long-term stored samples, 25 volatile compounds with significant classification contributions were screened using VIP>1. Detailed component information is presented in Table 4.

#### 3.2.5. Trends of Key Marker Compounds During Storage

**Figure 7:**
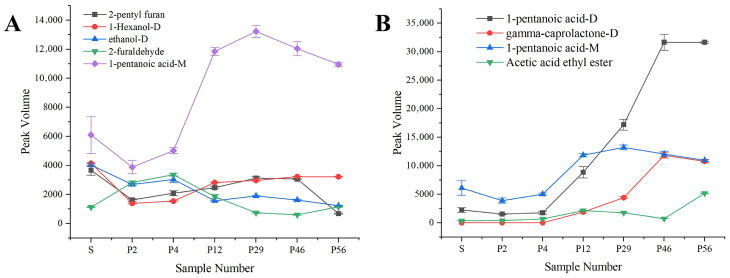
Expression patterns of key differential biomarkers (VIP > 2) across all samples: (**A**) S, P2; (**B**) P2, P4.

To better understand the chemical basis of flavor evolution during storage, key differential compounds with VIP > 2 were further analyzed. As shown in Figure 7A,B, 2-furaldehyde increased sharply after processing, contributing to the characteristic roasted aroma formed via Maillard reactions and thermal carbohydrate degradation, then gradually decreased during storage due to volatilization and further oxidation. Given its characteristic aroma profile of “sweet, woody, almond, bready”, this compound likely serves as a key contributor to the distinctive roasted flavor formed during processing. In contrast, 1-pentanoic acid, detected as both monomer and dimer, accumulated continuously over prolonged storage. Its fatty, slightly sour odor indicates a close association with lipid oxidation and the development of oxidative rancidity, highlighting its role as a major contributor to flavor deterioration. Both monomer and dimer signals of 1-pentanoic acid showed similar variation trends, confirming that the observed change reflects the accumulation of this compound rather than differences between ion forms. Its odor characteristics, including “fatty, decay, slightly sour, slightly sweet”, suggest that this compound is closely associated with lipid oxidation and degradation during long-term storage of coix seed and serves as a major contributor to oxidative rancidity odors.

Overall, PCA and OPLS–DA analyses confirmed stage-specific changes in volatile profiles and identified markers for differentiating storage stages. These selected markers were further used to construct a composite deterioration index, which integrates multiple chemically relevant variables into a single quantitative descriptor. These findings not only elucidate the phased impact of processing and storage on flavor quality at the chemical level but also provide clear chemical guidance for subsequent near-infrared spectroscopy-based key component correlation analysis and rapid non-destructive evaluation model development.

### 3.3. NIRS–GC–IMS Integrated Analysis

To achieve quantitative characterization of processing and storage changes in coix seeds, PLSR indices were first constructed based on GC–IMS data, using normalized peak volumes of key volatile compounds as independent variables and storage time or processing stage as dependent variables. All volatile compound peak volumes were normalized using the deuterated internal standard 2-Octanol-D to correct instrument fluctuations and reduce experimental variation. Compounds with VIP > 1 from OPLS–DA were selected as feature variables, capturing the synergistic evolution of multiple volatiles during processing and storage. These PLSR indices served as holistic flavor indicators, avoiding reliance on single compounds, and were subsequently used as *Y* variables in NIRS–PLSR models with preprocessed NIR spectra as *X*. Spectral preprocessing methods were systematically compared with optimize prediction accuracy and identify chemically meaningful wavelength regions.

#### 3.3.1. NIRS Prediction of Flavor Differences Between Raw and Processed Samples

To evaluate the ability of NIRS to capture chemical changes induced by processing, prediction models were developed based on the GC–IMS-derived processing index. The results demonstrate that NIRS can effectively distinguish raw and processed samples, reflecting substantial compositional transformations during roasting. The impact of different preprocessing methods on model performance was compared (Table 5). Five-fold cross-validation on the training set was used to optimize the PLSR model configuration, and the model’s predictive performance was assessed on an independent test set. Within 900–1700 nm, all preprocessing methods yielded high predictive accuracy, with SNVFD performing best ($R_{p}^{2}=0.985$, ${\mathrm{RMSE}}_{p}=0.484$, ${\mathrm{RPD}}_{p}=8.486$), indicating that derivative preprocessing enhances subtle spectral variations related to processing. In the 1300–2500 nm band, overall prediction performance further improved, with RAW and SG preprocessing yielding optimal results (${\mathrm{RMSE}}_{p}=0.272$, ${\mathrm{RPD}}_{p}=15.103$), suggesting that this band contains richer and more strongly correlated chemical information and demonstrates good robustness. These spectral regions are associated with overtone and combination vibrations of C–H, O–H, and C=O functional groups, which are closely linked to lipid structures and Maillard reaction products formed during roasting. This indicates that the spectral variation is directly related to underlying molecular transformations rather than empirical correlations alone.

**Figure 8:**
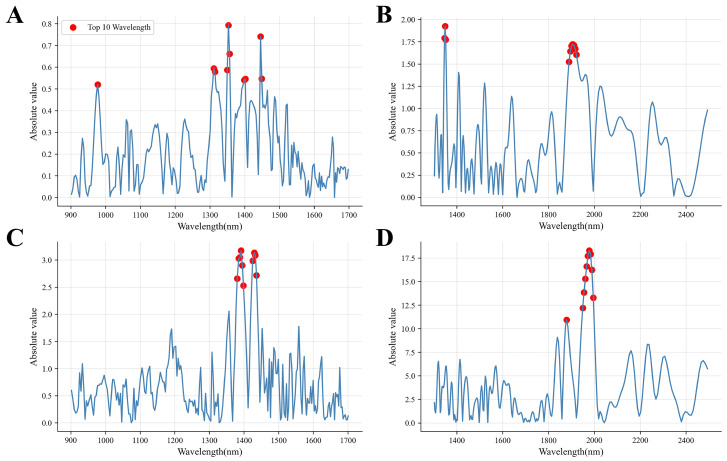
Key NIRS variable contributions in the GC–IMS-guided NIRS calibration model: (**A**) 900–1700 nm (flavor index); (**B**) 1300–2500 nm (flavor index); (**C**) 900–1700 nm (deterioration index); (**D**) 1300–2500 nm (deterioration index). NIRS: near-infrared spectroscopy; GC–IMS: gas chromatography–ion mobility spectrometry; PLSR: partial least squares regression.

To identify the spectral regions and compounds most influential for prediction, regression coefficients and VIP values were analyzed. The NIRS–PLSR regression coefficient distribution (Figure 8A,B) shows that high-contribution wavelengths are concentrated in the 1300–1500 nm and 1800–2000 nm regions, consistent with vibrational intervals of lipid- and carbonyl-related functional groups. Correspondingly, GC–IMS–PLSR VIP analysis identified 2-furaldehyde, 1-propanol-D, and 1-hexanol-D as the most influential volatiles, with 2-furaldehyde and 1-hexanol-D also exhibiting VIP>2 in OPLS–DA analysis. These compounds are recognized products of thermal processing and secondary reactions, such as the Maillard reaction and partial lipid degradation. The convergence between high-contribution NIR wavelengths and GC–IMS VIP-ranked compounds demonstrates that spectral variation is chemically anchored in processing-induced molecular transformations, providing mechanistic validation for this chemically guided multimodal approach.

#### 3.3.2. NIRS Prediction of Deterioration Index in Stored Processed Samples

To further evaluate the capability of NIRS for monitoring storage-induced deterioration, prediction models were developed based on the GC–IMS-derived deterioration index. In constructing this model, unprocessed raw samples were excluded, and only six processed samples (P2, P4, P12, P29, P46, P56) that underwent progressive changes over storage time were selected to characterize the gradually intensifying “rancid oil odor” feature of processed coix seed products during storage. The results indicate that NIRS can effectively track the progressive evolution of volatile compounds associated with rancid flavor development during storage. In Table 5, the impact of different preprocessing methods on model performance was compared. Within 900–1700 nm, SGFD preprocessing achieved the best performance ($R_{p}^{2}=0.923$, ${\mathrm{RMSE}}_{p}=1.241$, ${\mathrm{RPD}}_{p}=3.627$), indicating derivative processing enhances sensitivity to subtle spectral variations associated with O–H and C–H groups from lipid matrix and moisture-lipid interactions. In 1300–2500 nm, RAW preprocessing further improved prediction ($R_{p}^{2}=0.938$, ${\mathrm{RMSE}}_{p}=1.109$, ${\mathrm{RPD}}_{p}=4.060$), with high-contribution wavelengths at 1300–1500 nm and 1800–2000 nm corresponding to C–H, C=O, and O–H functional groups involved in lipid oxidation and secondary degradation. The superior sensitivity of this long-wave region highlights its utility for monitoring oxidative flavor changes, consistent with previous studies showing strong correlations between overtone NIR absorption and lipid degradation products.

To analyze spectral variables highly correlated with deterioration index prediction in the GC–IMS-guided NIRS calibration model, regression coefficients and variable importance were systematically evaluated. The NIRS–PLSR regression coefficient distribution (Figure 8C,D) shows that high-contribution wavelengths are concentrated in the 1300–1500 nm and 1800–2000 nm regions, consistent with vibrational intervals associated with lipid-related functional groups. This assignment is corroborated by the parallel accumulation of GC–IMS-identified oxidation markers, providing experimental cross-validation of the lipid oxidation interpretation. And VIP analysis identified 1-pentanoic acid-D, γ-caprolactone-D, and 2-hexanone-D as the most influential volatiles, which are well-known secondary oxidation products of fatty acids. This multimodal consistency indicates that the accumulation of aliphatic aldehydes and carboxylic acids identified by GC–IMS corresponds to increased regression coefficients in the 1300–1500 nm and 1900–2000 nm regions. This agreement provides quantitative cross-validation, linking spectral responses to lipid oxidation and secondary degradation. Although the NIRS–GC–IMS relationship is covariance-based, the temporal synchronicity under controlled moisture conditions, together with the specific correspondence between C–H and O–H vibrations and functional groups of aldehydes and carboxylic acids, supports a chemically meaningful association. Notably, the deterioration index is a dimensionless latent variable score with a total range of approximately 6 units across the 2–56 month storage period; the RMSEp of 1.109 for the optimal model thus corresponds to roughly 18% of this range, representing a prediction error that permits practical discrimination among samples with differing storage durations. Future correlation of the index with sensory panel scores will enable direct translation of prediction errors into sensory relevance. Such an approach provides practical potential for real-time quality monitoring in processed coix seed and other lipid-rich foods, supporting both shelf-life evaluation and storage condition optimization.

#### 3.3.3. Model Validation by Permutation Test

To validate model robustness and exclude the possibility of overfitting, permutation tests with 200 random iterations were performed for all four NIRS–PLSR calibration models. In each iteration, the response variable was randomly shuffled while the spectral matrix remained unchanged. The resulting $R^{2}$ and $Q^{2}$ values of the permuted models were recorded, and their regression lines against the correlation between original and permuted responses were computed.

As summarized in Table 6, the $Q^{2}$ intercepts of the permuted models were consistently negative, ranging from −0.220 to −0.465, which are well below the critical threshold of 0.05. These results confirm that the high predictive performance of the established models is statistically robust and not attributable to chance correlation.

## 4. Conclusions

In this study, we developed a chemically guided NIRS–GC–IMS calibration strategy for rapid evaluation of processing-induced flavor changes and storage deterioration in processed coix seeds. GC–IMS enabled identification of key volatile markers of thermal processing and oxidative degradation, which were integrated via PLSR to construct flavor and deterioration indices for NIRS calibration. Long-wave NIR spectra (1300–2500 nm) provided superior chemical relevance, capturing lipid oxidation and structural transformations, with regression coefficients aligning with VOC importance to validate chemical interpretability. Notably, the proposed strategy follows a two-phase framework: GC–IMS serves as a laboratory reference during one-time calibration, while routine quality assessment relies solely on portable NIRS devices. Future work will focus on external batch-wise validation across diverse origins, harvest years, and processing facilities, as well as instrument transferability across multiple spectrometers. In addition, we will explore nonlinear modeling approaches and evaluate model robustness under industrial operating conditions, including variable humidity scenarios and moisture compensation strategies, while integrating kinetic modeling of lipid oxidation and sensory analysis to establish operationally meaningful cutoff thresholds. Future investigations may also incorporate advanced interpretability tools such as SHAP analysis to further elucidate the contribution of individual spectral variables to model predictions. This enables reliable on-site monitoring of storage quality, shelf life, and flavor stability in processed coix seed products, and, following appropriate recalibration, can be extended to other cereal-based foods.

## Figures and Tables

**Table 1:** Classification results of NIRS spectral data from seven coix seed samples under different pretreatment methods and classification algorithms.

NIRS Range	Preprocessing Algorithm	Classification Algorithm	${\mathrm{Acc}}_{\mathrm{cv}}$	F1-${\mathrm{Score}}_{\mathrm{cv}}$	${\mathrm{Recall}}_{\mathrm{cv}}$	${\mathrm{Acc}}_{p}$	F1-${\mathrm{Score}}_{p}$	${\mathrm{Recall}}_{p}$
900–1700 nm	RAW	SVM	0.307	0.279	0.307	0.357	0.358	0.357
	RAW	KNN	0.489	0.476	0.489	0.400	0.388	0.400
	RAW	RF	0.571	0.563	0.571	0.571	0.553	0.571
	SGFD	SVM	0.629	0.618	0.629	0.671	0.661	0.671
	SGFD	KNN	0.500	0.492	0.500	0.471	0.461	0.471
	SGFD	RF	0.711	0.701	0.711	0.786	0.784	0.786
	MSCFD	SVM	0.693	0.684	0.693	0.729	0.725	0.729
	MSCFD	KNN	0.582	0.569	0.582	0.529	0.537	0.529
	**MSCFD**	**RF**	**0.800**	**0.797**	**0.800**	**0.829**	**0.822**	**0.829**
	SNVFD	SVM	0.707	0.696	0.707	0.729	0.725	0.729
	SNVFD	KNN	0.557	0.550	0.557	0.543	0.546	0.543
	SNVFD	RF	0.807	0.801	0.807	0.800	0.798	0.800
1300–2500 nm	RAW	SVM	0.364	0.319	0.364	0.457	0.419	0.457
	RAW	KNN	0.464	0.444	0.464	0.500	0.475	0.500
	RAW	RF	0.582	0.554	0.582	0.614	0.615	0.614
	SGFD	SVM	0.836	0.827	0.836	0.914	0.915	0.914
	SGFD	KNN	0.764	0.749	0.764	0.800	0.804	0.800
	SGFD	RF	0.889	0.888	0.889	0.914	0.914	0.914
	MSCFD	SVM	0.775	0.762	0.775	0.829	0.830	0.829
	MSCFD	KNN	0.725	0.709	0.725	0.771	0.765	0.771
	**MSCFD**	**RF**	**0.861**	**0.859**	**0.861**	**0.929**	**0.929**	**0.929**
	SNVFD	SVM	0.786	0.778	0.786	0.829	0.827	0.829
	SNVFD	KNN	0.732	0.715	0.732	0.786	0.775	0.786
	SNVFD	RF	0.846	0.844	0.846	0.900	0.898	0.900

Preprocessing algorithm: RAW: raw spectra; SGFD: SG and first derivative; MSCFD: MSC and first derivative; SNVFD: SNV and first derivative. Classification algorithm: SVM: support vector machine; KNN: k-nearest neighbor; RF: random forest. Acc: Accuracy; cv: 10-fold stratified cross-validation; p: independent test set. Bold values indicate the best performance for each NIRS range.

**Table 2:** Volatile components in raw and processed coix seed.

No.	Compound	CAS	MV	RI	Rt/s	Dt/ms
1	2-Butanone	78-93-3	72.1	926.8	190.557	1.24866
2	1-Hexanol-M	25917-35-5	102.2	1370.8	726.564	1.32859
3	Benzaldehyde-M	100-52-7	106.1	1501.3	983.51	1.1521
4	Butanol-M	71-36-3	74.1	1150.3	387.669	1.18446
5	1-Penten-3-ol	616-25-1	86.1	1165.3	409.006	0.94525
6	3-Methyl-2-butenal-M	107-86-8	84.1	1208.8	470.774	1.09303
7	1-Pentanol-D	30899-19-5	88.1	1264.9	557.804	1.51407
8	1-Pentanol-M	30899-19-5	88.1	1263.6	555.567	1.25549
9	2-methyl-2-hepten-6-one	129085-68-3	126.2	1354.5	699.927	1.17987
10	Benzaldehyde-D	100-52-7	106.1	1504.6	990.966	1.47342
11	1-Hexanol-D	25917-35-5	102.2	1374	731.866	1.64401
12	(E)-2-Heptenal-M	18829-55-5	112.2	1343	681.649	1.25902
13	Propanoic acid-M	79-09-4	74.1	1546.4	1092.198	1.10411
14	Propanoic acid-D	79-09-4	74.1	1545.9	1090.789	1.26024
15	2-propanone	67-64-1	58.1	836.9	155.686	1.1203
16	ethanol-M	64-17-5	46.1	963.5	212.36	1.04609
17	ethanol-D	64-17-5	46.1	946.5	201.986	1.13092
18	n-Pentanal-D	110-62-3	86.1	998.9	236.043	1.42467
19	n-Pentanal-M	110-62-3	86.1	1000.2	237.022	1.18157
20	2-Pentanone	107-87-9	86.1	995.3	233.499	1.37529
21	2-Pentanol	107-87-9	88.1	1127.6	357.464	1.44698
22	Butanol-D	71-36-3	74.1	1152.4	390.604	1.38209
23	1-Butanol, 3-methyl-D	123-51-3	88.1	1214.6	479.124	1.48346
24	1-Butanol, 3-methyl-M	123-51-3	88.1	1213.9	478.093	1.24562
25	2-Octanone-D	111-13-7	128.2	1303.3	622.26	1.76829
26	2-Octanone-M	111-13-7	128.2	1303.1	621.931	1.33829
27	1-octanal-M	124-13-0	128.2	1309.3	630.836	1.40099
28	(E)-2-Heptenal-D	18829-55-5	112.2	1342.2	680.307	1.6802
29	N,N-Dimethylformamide	68-12-2	73.1	1349.9	692.51	1.0322
30	(Z)-2-Penten-1-ol	1576-95-0	86.1	1345.7	685.914	0.94859
31	1-Hydroxy-2-propanone-M	116-09-6	74.1	1324.5	653.302	1.03813
32	1-nonanal-D	124-19-6	142.2	1401.5	779.959	1.9499
33	1-nonanal-M	124-19-6	142.2	1401.1	779.078	1.4737
34	Decanal-M	112-31-2	156.3	1489	955.649	1.54174
35	(E)-2-Pentenal-M	1576-87-0	84.1	1138.5	371.711	1.10727
36	3-Methyl-2-butenal-D	107-86-8	84.1	1210	472.525	1.36265
37	1-octanal-D	124-13-0	128.2	1309	630.356	1.8329
38	2-Methylpyrazine	109-08-0	94.1	1281.9	587.286	1.39461
39	α-terpinolene	586-62-9	136.2	1291.7	605.003	1.22387
40	(E,E)-2,4-heptadienal	4313-03-5	110.2	1482.1	940.609	1.1893
41	2-ethyl hexanol-M	104-76-7	130.2	1496.1	971.706	1.40738
42	(R/S)-linalool	78-70-6	154.3	1554.3	1112.266	1.22519
43	Propanal	123-38-6	58.1	770.2	134.763	1.14998
44	Ethyl formate	109-94-4	74.1	775.1	136.219	1.21612
45	2-Methylpropanal	78-84-2	72.1	778.4	137.19	1.28603
46	Butanal	123-72-8	72.1	876.8	169.713	1.28603
47	Acetic acid ethyl ester	141-78-6	88.1	887.9	173.839	1.34272
48	3-Methyl butanal	590-86-3	86.1	923.3	188.645	1.41452
49	α-Thujene-M	2867-05-2	136.2	1028	258.069	1.22364
50	1-Propanol-D	71-23-8	60.1	1043.9	270.953	1.2516
51	1-Propanol-M	71-23-8	60.1	1044.2	271.192	1.11246
52	(E)-2-Butenal-D	123-73-9	70.1	1054.7	280.064	1.20688
53	Acetic acid butyl ester-D	123-86-4	116.2	1075.7	298.638	1.62819
54	Acetic acid butyl ester-M	123-86-4	116.2	1075.5	298.475	1.24659
55	2-Hexanone-D	591-78-6	100.2	1085.6	307.882	1.51268
56	2-Hexanone-M	591-78-6	100.2	1081.9	304.383	1.1999
57	1-hexanal-M	66-25-1	100.2	1103.8	328.304	1.26659
58	1-hexanal-D	66-25-1	100.2	1110.3	335.974	1.57192
59	2-furaldehyde	98-01-1	96.1	1467.7	909.621	1.33676
60	1-pentanoic acid-D	109-52-4	102.1	1817.1	2049.132	1.51266
61	1-pentanoic acid-M	109-52-4	102.1	1818.8	2057.25	1.22492
63	γ-caprolactone-D	695-06-7	114.1	1676.5	1477.816	1.53881
64	γ-caprolactone-M	695-06-7	114.1	1678.1	1483.199	1.1941
65	3-Methyl butyric acid	503-74-2	102.1	1703.8	1574.722	1.22301
66	1-butanoic acid	107-92-6	88.1	1642	1364.049	1.16454
68	Acetic acid	64-19-7	60.1	1473.1	921.096	1.05115
69	1-Octen-3-ol-M	3391-86-4	128.2	1456.1	885.331	1.16205
70	Heptanol-M	111-70-6	116.2	1456.5	886.12	1.39646
71	Heptanol-D	111-70-6	116.2	1458.4	890.064	1.76457
72	1-Octen-3-ol-D	3391-86-4	128.2	1457.2	887.697	1.60135
73	2-ethyl hexanol-D	104-76-7	130.2	1493.9	966.735	1.80568
74	(E)-2-nonenal-D	18829-56-6	140.2	1514.6	1014.298	1.98017
75	(E)-2-nonenal-M	18829-56-6	140.2	1516.3	1018.317	1.41358
76	1-Octanol-D	111-87-5	130.2	1561.6	1131.53	1.88163
77	1-Octanol-M	111-87-5	130.2	1562.1	1132.87	1.469
80	(E)-2-octenal-D	2548-87-0	126.2	1422.8	819.43	1.82938
81	(E)-2-octenal-M	2548-87-0	126.2	1419.7	813.597	1.33605
82	2-Nonanone	821-55-6	142.2	1395.9	769.851	1.88611
85	1-Hydroxy-2-propanone-D	116-09-6	74.1	1323.2	651.288	1.2349
86	2,5-Dimethylpyrazine	123-32-0	108.1	1335.1	669.327	1.11612
87	2-pentyl furan	3777-69-3	138.2	1237.8	513.868	1.25659
88	γ-Terpinene-M	99-85-4	136.2	1248.7	531.177	1.22356
89	γ-Terpinene-D	99-85-4	136.2	1248.2	530.289	1.71298
90	(E)-2-hexenal	6728-26-3	98.1	1228.7	499.907	1.52326
91	Pyrazine-M	290-37-9	80.1	1219.6	486.44	1.05057
92	Pyrazine-D	290-37-9	80.1	1220.8	488.123	1.27985
93	Butanoic acid butyl ester	109-21-7	144.2	1222.3	490.398	1.83167
94	(+)-limonene	5989-27-5	136.2	1192.9	448.6	1.22385
95	2-Heptanone	110-43-0	114.2	1185	438.107	1.63928
96	Butyl 2-propenoate	1214-39-7	128.2	1176.9	426.367	1.69287
97	Amyl acetate	628-63-7	130.2	1170.5	416.762	1.76904
99	2-Methylbutyl acetate	624-41-9	130.2	1143.2	377.985	1.72672
101	(E)-2-Pentenal-D	1576-87-0	84.1	1136.3	368.736	1.36849
103	1-Propanol, 2-methyl	78-83-1	74.1	1105.5	330.315	1.17526
104	2-Methylthiophene	554-14-3	98.2	1112.4	338.497	1.02999
105	2-methyl-3-pentanol	565-67-3	102.2	1123.3	352.016	1.56594
106	Acetaldehyde	75-07-0	44.1	678.8	110.611	0.96377
109	Decanal-D	112-31-2	156.3	1488.1	953.679	2.06242
110	Methyl propanoate	554-12-1	88.1	857.3	162.694	1.32719
111	(E)-2-Butenal-M	123-73-9	70.1	1056.4	281.575	1.03802
112	α-Thujene-D	2867-05-2	136.2	1025.6	256.179	1.67379
115	3-Pentanol	584-02-1	88.1	1115.7	342.56	1.42313
116	(E)-2-Methyl-2-butenal-D	497-03-0	84.1	1104.4	329.029	1.35808
117	(E)-2-Methyl-2-butenal-M	497-03-0	84.1	1101.5	325.63	1.09961
121	β-Thujene	3387-41-5	136.2	1113.5	339.904	1.2223
122	β-myrcene	123-35-3	136.2	1159.8	401.037	1.22349
123	2-methyl-1-butanol	137-32-6	88.1	1214.5	478.964	1.23123

CAS: Chemical Abstracts Service registry number; MV: monomer volatile; RI: retention index; Rt: retention time; Dt: drift time; The suffixes “-M” and “-D” denote monomer and dimer forms of the same volatile compound, respectively, as detected by GC–IMS.

**Table 3:** Differentiation markers for raw products (S) and processed products (P2).

No.	Compound	CAS	Sensory Descriptions
7	1-Pentanol-D	30899-19-5	balsamic
8	1-Pentanol-M	30899-19-5	balsamic
**11**	**1-Hexanol-D**	**25917-35-5**	**fresh, fruity, wine, sweet, green**
14	Propanoic acid-D	79-09-4	yogurt, vinegar
**17**	**ethanol-D**	**64-17-5**	**aromaticity**
20	2-Pentanone	107-87-9	acetone, fresh, sweet fruity, wine
22	Butanol-D	71-36-3	wine
28	(E)-2-Heptenal-D	18829-55-5	spicy, green vegetables, fresh, fatty
31	1-Hydroxy-2-propanone-M	116-09-6	spicy, green vegetables, fresh, fatty
36	3-Methyl-2-butenal-D	107-86-8	fruity
37	1-octanal-D	124-13-0	aldehyde, waxy, citrus, orange, fruity, fatty
38	2-Methylpyrazine	109-08-0	nutty, moldy, roast, earthy
50	1-Propanol-D	71-23-8	alcohol, pungent
58	1-hexanal-D	66-25-1	fresh, green, fat, fruity
**59**	**2-furaldehyde**	**98-01-1**	**sweet, woody, almond, bready**
60	1-pentanoic acid-D	109-52-4	fatty, decay, slightly sour, slightly sweet
**61**	**1-pentanoic acid-M**	**109-52-4**	**fatty, decay, slightly sour, slightly sweet**
64	γ-caprolactone-M	695-06-7	herb, coconut, sweet, coumarin, tobacco
68	Acetic acid	64-19-7	spicy
70	Heptanol-M	111-70-6	herb
71	Heptanol-D	111-70-6	herb
75	(E)-2-nonenal-M	18829-56-6	herb
76	1-Octanol-D	111-87-5	citrus, sweet, herbs, waxy, rose, mushroom
77	1-Octanol-M	111-87-5	citrus, sweet, herbs, waxy, rose, mushroom
80	(E)-2-octenal-D	2548-87-0	fresh cucumber, fatty, green herbal, banana, green leaf
85	1-Hydroxy-2-propanone-D	116-09-6	pungent, caramel, fresh
**87**	**2-pentyl furan**	**3777-69-3**	**bean, fruity, earthy, green, vegetable**
88	γ-Terpinene-M	99-85-4	oil, wood, terpenes, lemon, lime, herbs
90	(E)-2-hexenal	6728-26-3	green, banana, fat
95	2-Heptanone	110-43-0	pear, banana, fruity, slight medicinal fragrance
96	Butyl 2-propenoate	1214-39-7	pungent, fruity
101	(E)-2-Pentenal-D	1576-87-0	potato, peas

Bold indicates marker compounds with VIP > 2.

**Table 4:** Differentiation markers for processed products (P2) and processed products (P56).

No.	Compound	CAS	Sensory Descriptions
11	1-Hexanol-D	25917-35-5	fresh, fruity, wine, sweet, green
13	Propanoic acid-M	79-09-4	yogurt, vinegar
14	Propanoic acid-D	79-09-4	yogurt, vinegar
17	ethanol-D	64-17-5	aromaticity
20	2-Pentanone	107-87-9	acetone, fresh, sweet fruity, wine
25	2-Octanone-D	111-13-7	moldy, ketone, milk, cheese, mushroom
32	1-nonanal-D	124-19-6	rose, citrus, strong oily
37	1-octanal-D	124-13-0	aldehyde, waxy, citrus, orange, fruity, fatty
38	2-Methylpyrazine	109-08-0	nutty, moldy, roast, earthy
**47**	**Acetic acid ethyl ester**	**141-78-6**	**fresh, fruity, sweet, grassy**
55	2-Hexanone-D	591-78-6	fruity, fungal, meaty, buttery
57	1-hexanal-M	66-25-1	fresh, green, fat, fruity
59	2-furaldehyde	98-01-1	sweet, woody, almond, bready
**60**	**1-pentanoic acid-D**	**109-52-4**	**fatty, decay, slightly sour, slightly sweet**
**61**	**1-pentanoic acid-M**	**109-52-4**	**fatty, decay, slightly sour, slightly sweet**
**63**	**γ-caprolactone-D**	**695-06-7**	**herb, coconut, sweet, coumarin, tobacco**
64	γ-caprolactone-M	695-06-7	herb, coconut, sweet, coumarin, tobacco
66	1-butanoic acid	107-92-6	strong acetic acid, cheese, butter, fruity
80	(E)-2-octenal-D	2548-87-0	fresh cucumber, fatty, green herbal, banana, green leaf
82	2-Nonanone	821-55-6	fresh, sweet, green, herb
88	γ-Terpinene-M	99-85-4	oil, wood, terpenes, lemon, lime, herbs
95	2-Heptanone	110-43-0	oil, wood, terpenes, lemon, lime, herbs
96	Butyl 2-propenoate	1214-39-7	pungent, fruity

Bold indicates marker compounds with VIP > 2.

**Table 5:** NIRS–GC–IMS PLSR prediction performance.

Index Type	NIRS Range	Preprocessing Algorithm	$R_{\mathrm{cv}}^{2}$	${\mathrm{RMSE}}_{\mathrm{cv}}$	${\mathrm{RPD}}_{\mathrm{cv}}$	$R_{p}^{2}$	${\mathrm{RMSE}}_{p}$	${\mathrm{RPD}}_{p}$
**Flavor Index**	900–1700 nm	RAW	0.941	0.992	4.135	0.930	1.057	3.885
		SG	0.948	0.931	4.406	0.930	1.055	3.892
		MSC	0.975	0.642	6.393	0.972	0.668	6.148
		SNV	0.972	0.687	5.973	0.977	0.603	6.810
		SGFD	0.945	0.956	4.293	0.929	1.067	3.847
		MSCFD	0.977	0.612	6.700	0.977	0.603	6.811
		**SNVFD**	**0.982**	**0.542**	**7.577**	**0.985**	**0.484**	**8.486**
	1300–2500 nm	RAW	0.996	0.258	15.894	0.995	0.277	14.793
		**SG**	**0.996**	**0.254**	**16.175**	**0.995**	**0.272**	**15.103**
		MSC	0.986	0.481	8.538	0.990	0.391	10.486
		SNV	0.991	0.387	10.608	0.994	0.311	13.205
		SGFD	0.990	0.408	10.057	0.987	0.450	9.113
		MSCFD	0.979	0.589	6.968	0.985	0.489	8.396
		SNVFD	0.985	0.503	8.164	0.979	0.586	7.010
**Deterioration** **Index**	900–1700 nm	RAW	0.880	1.467	2.898	0.899	1.419	3.173
		SG	0.879	1.475	2.883	0.895	1.445	3.116
		MSC	0.859	1.592	2.672	0.860	1.667	2.700
		SNV	0.901	1.332	3.191	0.875	1.576	2.857
		**SGFD**	**0.900**	**1.341**	**3.171**	**0.923**	**1.241**	**3.627**
		MSCFD	0.845	1.668	2.549	0.877	1.568	2.872
		SNVFD	0.891	1.399	3.039	0.911	1.335	3.371
	1300–2500 nm	**RAW**	**0.935**	**1.079**	**3.940**	**0.938**	**1.109**	**4.060**
		SG	0.936	1.074	3.960	0.938	1.116	4.035
		MSC	0.914	1.244	3.419	0.935	1.141	3.946
		SNV	0.923	1.176	3.614	0.930	1.183	3.806
		SGFD	0.921	1.196	3.556	0.925	1.221	3.687
		MSCFD	0.910	1.275	3.335	0.922	1.245	3.617
		SNVFD	0.915	1.238	3.434	0.929	1.191	3.779

Performance metrics: $R_{\mathrm{cv}}^{2}$, ${\mathrm{RMSE}}_{\mathrm{cv}}$, ${\mathrm{RPD}}_{\mathrm{cv}}$—coefficient of determination, root mean square error, and residual prediction deviation for training set (5-fold cross-validation); $R_{p}^{2}$, ${\mathrm{RMSE}}_{p}$, ${\mathrm{RPD}}_{p}$—corresponding metrics for independent test set. Preprocessing abbreviations (RAW, SG, MSC, SNV, SGFD, MSCFD, SNVFD) are as defined in Table 1. Bold values indicate the optimal results for each model.

**Table 6:** Permutation test summary for NIRS–PLSR models of flavor and deterioration indices.

Index Type	NIRS Band	Original $R^{2}$	Original $Q^{2}$	$Q^{2}$ Intercept
Flavor Index	900–1700 nm	0.984	0.984	−0.429
	1300–2500 nm	0.900	0.900	−0.407
Deterioration Index	900–1700 nm	0.996	0.996	−0.465
	1300–2500 nm	0.935	0.935	−0.220

## Data Availability

The datasets presented in this article are not readily available because they are part of an ongoing study involving the development of a spectral database and due to the large size of the spectral datasets. Requests to access the datasets should be directed to the corresponding author.

## Abbreviations

The following abbreviations are used in this manuscript:

NIRS	Near-infrared spectroscopy
GC–IMS	Gas chromatography–ion mobility spectrometry
